# Project HERO: a randomized trial of Tai Chi qigong versus intensity-matched exercise and usual care for fatigue in older male cancer survivors

**DOI:** 10.1186/s12906-025-04988-7

**Published:** 2025-07-04

**Authors:** Anita Y. Kinney, Jinghua An, Yong Lin, Samuel Tundealao, Biren Saraiya, Shou-En Lu, Dolores D. Guest, Elizabeth M. Harding, Fabiano Amorim, Emily Heidt, Evelyn Arana-Chicas, Chunxia Chen, Tawny Boyce, Isaac Y. Kim, Wadih Arap, Cindy K. Blair, Michael R. Irwin

**Affiliations:** 1 Department of Biostatistics and Epidemiology, Rutgers University School of Public Health, Piscataway, NJ USA; 2 Rutgers Cancer Institute, New Brunswick, NJ USA; 3 Division of Medical Oncology, Section of Solid Tumors, Robert Wood Johnson Medical School, Rutgers University, New Brunswick, NJ USA; 4 University of New Mexico Comprehensive Cancer Center, Albuquerque, NM USA; 5 Department of Health, Exercise and Sports Sciences, University of New Mexico, Albuquerque, NM USA; 6 Division of Medical Oncology, Section of Behavioral Science, Robert Wood Johnson Medical School, Rutgers University, New Brunswick, NJ USA; 7 Yale New Haven Hospital, New Haven, CT USA; 8 Rutgers Cancer Institute, Newark, NJ USA; 9 Division of Hematology and Oncology, Department of Medicine, Rutgers New Jersey Medical School, Newark, NJ USA; 10 Department of Internal Medicine, University of New Mexico, Albuquerque, NM USA; 11 Department of Psychiatry, David Geffen School of Medicine, University of California, Los Angeles, CA USA; 12 Cousins Center for Psychoneuroimmunology, Semel Institute for Neurosciences, University of California-Los Angeles, Los Angeles, CA USA

**Keywords:** Fatigue, Cancer, Tai Chi, Qigong, Mind-Body therapies, Complementary therapy

## Abstract

**Background:**

Fatigue is often one of the most commonly reported symptoms in older male cancer survivors, but it is also one of the least understood cancer-related symptoms. Fatigue is associated with psychological distress, disruptions in sleep quality, and impairments in health-related quality of life. Thus, elective treatments for fatigue in older male cancer survivors represent a current unmet need. Prior research has shown that Tai Chi Qigong (TCQ), a mind-body exercise intervention, can improve physical and emotional health. Therefore, we compared the efficacy of Tai Chi Qigong (TCQ) versus exercise intensity-matched (EIM) and usual care in older, male cancer survivors with fatigue.

**Methods:**

We conducted a three-arm, single-blind randomized controlled trial where older (55 + years), male cancer survivors with fatigue participated in usual care or one of two supervised group exercise programs: TCQ or EIM twice weekly for 12 weeks. Participants were followed up for 12 months. The primary outcome was patient-reported fatigue at 3-months post-intervention.

**Results:**

A cohort of men (*n* = 113) were enrolled (mean age: 69.1 (±7.0) years. In the primary outcome analysis, there were no significant within-arm or between-arm differences in fatigue (*p-*value, NS). However, the TCQ and EIM arms showed significant within-arm improvement in fatigue immediately post-intervention (*p-*value < 0.05). There were no differences in class attendance for either TCQ or EIM, with an average attendance rate of 78.4% and 76.8%, respectively.

**Conclusion:**

We found no significant or clinically meaningful improvements in fatigue for TCQ or EIM relative to usual care at the 3-month follow-up. However, significant improvements in fatigue were observed immediately after completion of the 12-week TCQ and EIM programs. This study suggests that TCQ and light intensity activity may lead to improvements in fatigue immediately after the group exercise program among older, fatigued male cancer survivors. However, the observed improvements did not persist beyond the program, suggesting that long-term maintenance may be required. Further testing is warranted in larger trials that include strategies to sustain both the behavior and the effects.

**Trial registration:**

This study was registered at the NIH clinical trial registry on November 17, 2017 (NCT03345563).

**Supplementary Information:**

The online version contains supplementary material available at https://doi.org/10.1186/s12906-025-04988-7.

## Background

Cancer-related fatigue (CRF) is a common and distressing symptom experienced by many cancer survivors. The National Comprehensive Cancer Network (NCCN) describes CRF as “a persistent, subjective feeling of exhaustion, physical, emotional, or cognitive tiredness that is not proportional to recent activity and interferes with normal functioning” [1]. The prevalence of CRF ranges widely from 25 to 95%, depending on the method of assessment, patient population, and type of treatment [2]. Approximately one-third of all cancer survivors who complete treatment will continue to experience fatigue for months to years [3, 4]. Despite the high prevalence of fatigue, it remains underdiagnosed and undertreated and is one of the least understood cancer-related symptoms [5, 6]. Among older cancer survivors, CRF can be exacerbated by lack of physical activity, aging-related comorbidities, and increasing limitations in physical, mental, and social functioning [1, 4, 7–11]. These factors, along with CRF, may influence older survivors’ aptitude for physical activity because of worsening functional limitations and quality-of-life impairments [12, 13].

Research has shown that exercise and other non-pharmaceutical interventions, such as mindfulness-based interventions during and/or after cancer treatment, improves CRF and related symptoms [14–17]. Although recommendations from the American Society of Clinical Oncology (ASCO) and the NCCN for managing fatigue recommend exercise, it is not generally included in survivorship plans [1, 18]. Moreover, evidence to inform health promotion survivorship guidelines for treating fatigue in male cancer survivors shows that both aerobic exercise and resistance training can reduce CRF [19–23]. Findings from a recent meta-analysis suggest that exercise interventions had a more favorable effect on improving CRF in female cancer survivors than male cancer survivors [23]. Finally, many of the studies evaluating the impact of exercise on CRF incorporated moderate to high-intensity types of physical activity [24].

The Biopsychosocial Model posits that mindfulness-based interventions that foster physical activity, relaxation, physiological adaptive changes, and psychological well-being may lead to improvements in fatigue [25]. Meditative movement practices such as Tai Chi and Qigong (TCQ) incorporate movement or postures with a focus on breathing and a meditative state to attain deep relaxation states. However, the level of exertion of these types of practices can vary considerably and depend on the type of practice or specific exercises. Most Tai Chi movements or postures are considered to have a low level of exercise intensity [26]. Thus, these practices may be easier to engage in than resistance training or more strenuous aerobic exercises for persons with CRF. Furthermore, Tai Chi and Qigong have been effectively used in elderly and medically compromised populations, including cancer patients [17, 27, 28]. There is a growing body of evidence that meditative movement practices such as Tai Chi and Qigong lead to improvements in CRF; however, studies involving Tai Chi and Qigong separately or combined (TCQ) primarily involved women with breast cancer, with some focused on patients with colorectal, lung, and prostate cancer; few trials focused on male cancer survivors [29–32]. Two randomized pilot trials evaluated the impact of TCQ on CRF in men with prostate cancer; one study found improvements in fatigue [26], while the other study found no appreciable effect of TCQ on fatigue [33]. These conflicting reports underscore the need for more trials of the impact of TCQ on CRF in male cancer survivors. Moreover, previous research on TCQ’s effects in cancer patients often had a relatively short follow-up period and lacked an active, exercise intensity-matched control group; hence, the current study design attempted to address the limitations [26, 30, 32, 33].

The primary objective of the Health Recovery and Empowerment Outcomes (HERO) randomized controlled trial was to determine the efficacy of a standardized TCQ intervention for reducing fatigue (primary outcome) compared to an exercise intensity-matched (EIM) intervention and usual care among male cancer survivors. TCQ differs from traditional exercise interventions by including a focus on deep breathing techniques, synchronized, and rhythmic movements, specific postures, and meditation to induce relaxation. Further, TCQ is comprised of dynamic mental and physical exercises where the muscles are relaxed and can induce a meditative state while the EIM intervention primarily focused on static exercises with muscles tensed with no purposeful meditative components. Both TCQ and EIM included stretching and eccentric movements.

We hypothesized that men in the TCQ group would have greater improvements in fatigue levels compared to those in the EIM group (active control) arm and usual care (control) arm 3 months following 12 weeks of group based TCQ. We also explored whether intervention effects were durable at the 12- month follow-up. This report adheres to CONSORT guidelines for reporting randomized trials of nonpharmacologic treatments [34].

## Methods

### Study design

The study design was informed by the high-priority research recommendations on cancer-related fatigue, as outlined by the Clinical Trials Planning Meeting convened by the National Cancer Institute [35]. The study protocol has been described in detail [36]. This 3-arm parallel group randomized controlled trial was approved by Institutional Review Boards at Rutgers University and the University of New Mexico. Potentially eligible men were recruited in clinics at Rutgers Cancer Institute and the University of New Mexico Comprehensive Cancer Center clinics and community settings in New Brunswick and Albuquerque metropolitan areas. After completing informed consent and the baseline assessment, participants were randomly assigned with a 2:2:1 allocation as per a computer-generated randomization schedule to one of three groups: (1) TCQ, (2) EIM, and (3) usual care. Trained research staff enrolled participants and informed them of their study arm assignment. Participants in the TCQ or EIM arms were not informed whether their group represented experimental or control conditions. Men participated in their assigned concurrent TCQ and EIM group interventions for 12 weeks with 12 months of follow-up. Furthermore, we avoided the use of the terms Tai Chi and Qigong for participant recruitment and research materials such as the participant written consent forms, recruitment materials, assessments, and intervention implementation. Instead, we referred to the intervention groups as follows: TCQ intervention was referred to as body-mind training (BMT) and the exercise intensity-matched condition was referred to as body training (BT). This was done to help to reduce bias by minimizing participants’ expectations and/or perceived differences between the three groups. Study recruitment occurred between June 2017 and November 2021. Interventions occurred between August 2017 and February 2022. Data analysts were blinded to participants’ study arm allocation.

### Participants

The target population included older male cancer survivors who reported fatigue. Eligibility criteria included: (1) age *≥* 55 years; (2) pathologically confirmed local or regional stages of the following cancers: colorectal, lung oral/pharynx, small intestine, soft tissue, thyroid, non-muscle invasive bladder, kidney, or renal pelvis; or prostate cancer– local, regional, or with elevated prostate-specific antigen (PSA) (this eligibility criteria was expanded beyond prostate cancer due to recruitment challenges during COVID-19); (3) in cancer remission or stable disease; (4) completed cancer treatment except for androgen deprivation therapy; (5) if on androgen deprivation therapy, must have been taking it for four or more months; (6) scored *≤* 13 on the Medical Health Outcome Study Short Form Vitality/Fatigue subscale (SF-36 Vitality scale) [37] or a score of *≥* 9 the Patient-Reported Outcomes Measurement Information System Fatigue scale (PROMIS Fatigue scale) [38]; (7) engaged in < 150 min of moderate-to-vigorous exercise/week within the past 3 months. Participants were asked to answer 5 questions eliciting information about the type, frequency, amount, and intensity of activity. Individuals were provided with definitions for mild, moderate and vigorous activity; standard descriptions of mild, moderate and high intensity exercise were provided; (8) ability to speak and read English and (9) answered “no” to the question: “Have you ever been told by a doctor that you should not engage in mild to moderate exercise or physical activity for any reason?” Men were considered not eligible and therefore excluded from the study if: (1) their Patient Health Questionnaire-9 (PHQ-9) score was *≥* 12, indicating moderate-to-severe depression; (2) their Karnofsky performance status score (KPS) was 50% or below; (3) they reported uninterrupted engagement in a mindfulness-based intervention(s) in the prior year for two or more times a week over two or more months; and/or (4) currently receiving cancer treatment other than hormonal ADT.

### Interventions

***Tai Chi Qigong (TCQ)***. The manualized TCQ sessions, led by an experienced Qigong Master or one of her/his certified instructors, were held twice a week for 12 weeks. Each session included: (1) warm up and a review of TCQ principles; (2) meditation with TCQ movement; (3) breathing techniques; and (4) relaxation procedures. The principles of TCQ generally include (1) focusing on controlling and increasing energy, distributing throughout the body; (2) focusing on breath for body relaxation and meditation; (3) integrating awareness, breath, alignment, centering, and posture; (4) rooting the soles of the feet to the ground, with knees bent in a low stance, and primary focus of awareness within lower abdomen; (5) emphasizing a soft, relaxed posture, rather than a hard, tense posture; and (6) practicing moves with a quiet and open mind as much as possible.

Sessions began with a five-minute meditative focus on the breath, followed by sitting exercises, then standing movements (19 types of movements total), and ended with a final five-minute meditative focus on the breath. As the study progressed, a larger proportion of time was spent performing the standing movements than the sitting exercises. An eccentrically biased muscular focus was integrated into the standing movements to increase the intensity of the intervention. Eccentrically biased movements use body weight as resistance and target eccentric muscle contractions where the muscle lengthens. This entailed deeper squatting positions in the standing movements. Each class lasted 60 min, taking place twice per week, and was supplemented with home-based practice using an instructional DVD and handouts illustrating the specific movements and poses to help with their independent practice. Men were encouraged to practice at home for at least 30 min (preferably 60 min) sessions 3 days per week during the 12-week intervention period and during the 12-month follow-up period. A description of the movements and postures has been previously published [36].

***Exercise Intensity Matched (EIM) Intervention***. The manualized EIM control group received a movement-based, intensity-matched exercise group and controlled for non-specific factors such as social attention, contact time, cardiopulmonary exertion and group setting. Additionally, this intervention accounted for stretching (e.g., extending arms upward and outward), repetitive and eccentric movements (e.g., bending and moving back and forth) and intensity of physical activity. The EIM intervention was developed by two exercise physiologists (F.A. and E.M.H.). They designed the exercise program simulating the TCQ movement pattern in muscle action (eccentric, concentric and isometric), posture (seated or standing), number of sets and repetitions. Before implementing the trial, the intensity of the two exercise interventions was established in a small group of persons. The oxygen consumption through indirect calorimetry (Parvomedics, Trueone 2400, Sandy, Utah) heart rate Polar M400 (Polar Electro Oy, Kempele, Finland), and rate of perceived exertion using the Borg Rating of Perceived Exertion Scale [39] were assessed: no differences were found between the TCQ and EIM sessions.

Like the TCQ classes, the EIM classes met twice a week for 60 min each session for 12 weeks. EIM participants were provided with an instructional DVD and handouts illustrating the specific movements (e.g., arm raises, chair stands, pelvic rocking, side stretches) to help with their independent practice and encouraged to engage in home practice for at least 30 min (preferably 60 min) each time, 3 days per week. As with the TCQ arm, quality control measures to ensure intervention fidelity were described [36].

As with TCQ, EIM in-person group classes were held at Rutgers University, New Brunswick, New Jersey or at a community site in Albuquerque, New Mexico until the beginning of the COVID-19 pandemic in March 2020 when classes needed to be expeditiously converted to virtual synchronous group sessions. Once the COVID restrictions loosened in September 2020, classes were again held in-person and/or virtually depending on participants’ preference to enhance the ability to recruit and retain participants during this challenging time. All three groups were treated the same way during the COVID-19 pandemic.

#### Usual care (UC)

Participants in the usual care control group received care as normal and completed the same baseline and follow-up assessments as participants in the TCQ and EIM groups.

### Data collection and measures

Baseline demographic and clinical data were assessed by self-report and medical records. Fatigue, the primary outcome, was measured with the Functional Assessment of Chronic Illness-Fatigue (FACIT-F) Scale [40, 41] at baseline (within 2 weeks before the TCQ/EIM interventions), 6 weeks mid-intervention, 1-week post-intervention, and 3-month and 12-month post-intervention. The FACIT-F is frequently used to measure self-reported fatigue and its impact on daily activities among cancer survivor populations. This 13-item scale assesses level of fatigue during usual activities over the past 7 days, with higher scores indicating less fatigue (score range 0–52; α = 0.86–0.87) [38].

Participants’ perceptions about the credibility and effectiveness of an intervention has been strongly correlated with their engagement and the likelihood of positive outcomes [40]. Thus, treatment credibility and expectations were assessed during the first week of class, and at 6 weeks mid-intervention and 1 week post-intervention by using the 6-item credibility/expectancy scale modified for a group-based intervention with health and well-being [42]. This scale assesses both an expectancy and credibility factor and includes items about participants’ thoughts and feelings about the likelihood that the intervention will have the desired effect on health and well-being, as well as expectations about the size of the effects. The scale has good internal consistency (α = 0.79–0.81) and high test-retest reliability (0.75–0.82) [40].

The Borg scale is a widely used and reliable measure of exercise intensity [39]. To monitor and guide intensity, TCQ and EIM participants were asked to complete the Borg scale immediately after class and rate their overall feeling of exertion during the class. Intervention fidelity for TCQ and EIM was assessed by having a research assistant attend each session and use intervention fidelity checklists to confirm adherence to the study protocol. All sessions were rated as adherent. Class attendance was also assessed.

### Statistical analysis

The statistical analyses were performed on an intent-to-treat basis. The primary outcome is the FACIT-F score change from baseline to 3-months, while the secondary outcomes were FACIT-fatigue score change at 6-week mid-intervention, and at 1-week post intervention and 12-month post intervention. An increase of > 3 points in the FACIT-F was considered a clinically meaningful improvement in fatigue [43–45]. Mixed model analysis was used to analyze the score changes with FACIT-fatigue score as the dependent variable; intervention group, time, and intervention group-by-time interaction were included as fixed effects independent variables. Participant was included as a random effect to account for the within-person correlation in the repeatedly measured outcomes. Linear contrasts were used to estimate the score changes within each treatment group and compared between groups, with 95% confidence intervals (CIs). Missing data were imputed by using the fully conditional specification (FCS) multiple imputation approach, assuming missing at random [46]. Twenty-five imputed datasets were generated based on age, race, ethnicity, marital status, education attainment, household income, cancer type (prostate vs. other), and currently taking hormonal ADT or not. Statistical analysis was performed on each imputed dataset and combined by using the Rubin’s rule via Proc MI and Proc Mianalyze in SAS to check the robustness of the findings [47]. The effect of the interventions in men with prostate cancer as a sensitivity analysis was also analyzed. For all analyses, a two-sided *p*-value < 0.05 was considered statistically significant. All analyses were performed in SAS version 9.4 (SAS Institute Inc, Cary, NC).

### Sample size and power

A target sample size of 123 subjects was determined for 3 treatment groups (49, 49, 25 subjects with 2:2:1 allocation ratio for TCQ, EIM, and UC groups, respectively) to test a between-group difference in fatigue at 3 months post-intervention (primary endpoint) with an effect size Cohen’s d = 0.63 for comparing TCQ vs. EIM and Cohen’s d = 0.78 for TCQ vs. UC, with alpha = 0.025 (two-sided after Bonferroni adjustment) and power = 80% for each comparison. A total of 166 participants was determined after accounting for approximately 25% attrition. Interruptions due to the COVID-19 pandemic adversely impacted enrollment; the accrual goal was not reached, and 113 participants were enrolled in the study (45, 43, 25 participants for TCQ, EIM, and UC, respectively [48–50].

## Results

**Fig. 1:**
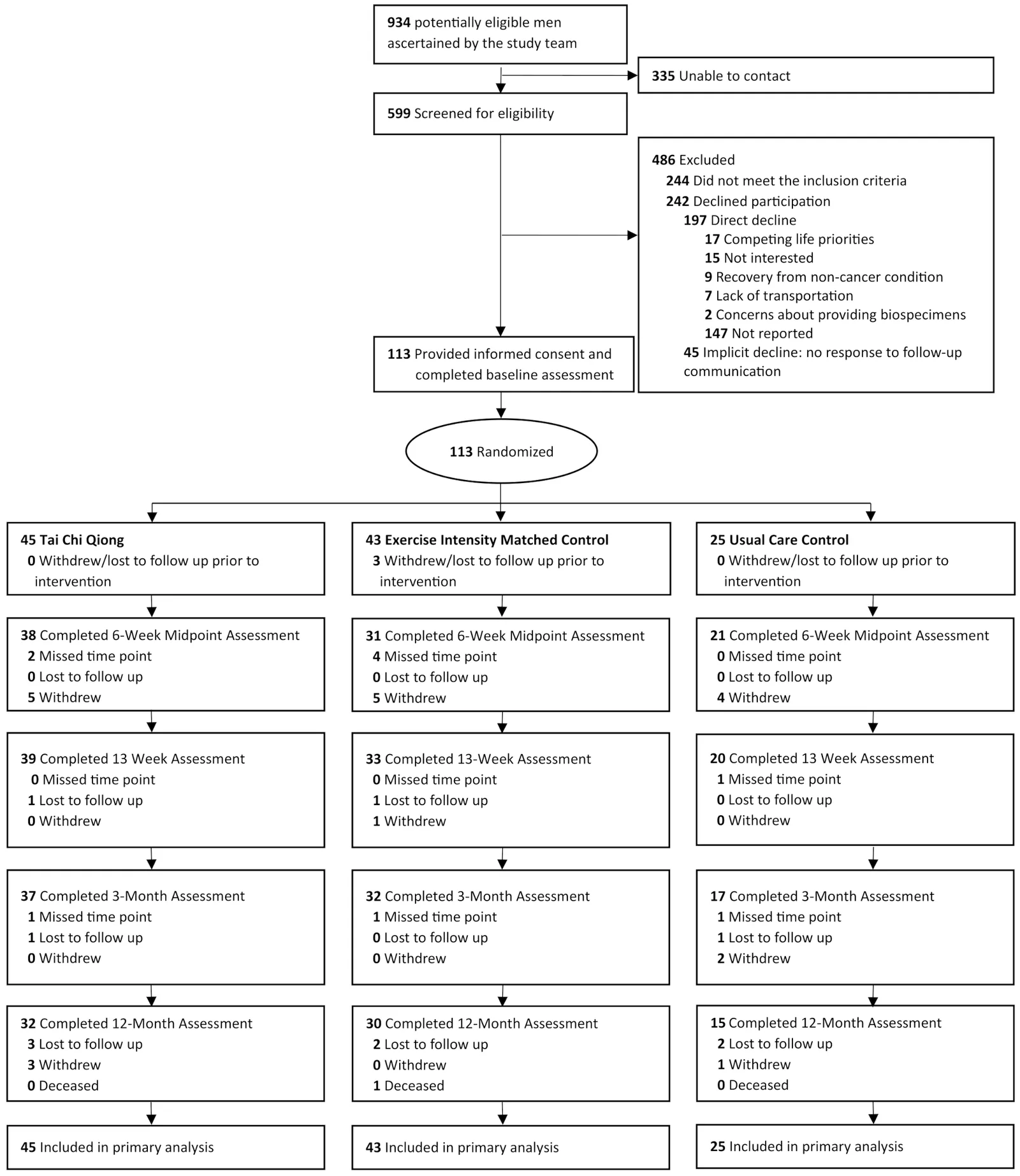
HERO Trial CONSORT Diagram

In total, 113 male cancer survivors were enrolled in the trial; all 113 participants were included in the analysis. The study enrollment, random arm assignment, and retention data are shown in Fig. 1. The overall retention rate at 3 months was 76.0%. Table 1 summarizes demographic and clinical characteristics for the total sample and by intervention arm. Among the 113 participants, the average age was 69.1 (±7.0) years, and most patients were non-Hispanic (88.5%), White (77.0%), and married or in a domestic partnership (76.8%). The median time since cancer diagnosis was 4 years. 87% of participants had a diagnosis of prostate cancer. The demographic characteristics of participants with prostate cancer (Supplemental Table 1) showed a similar distribution compared with the overall sample.

**Table 1:** Sociodemographic and clinical characteristics of participants by study arm

Study Arm	All (N = 113)	TCQ (N = 45)	EIM (N = 43)	UC (N = 25)	*P* value^a^
	*n* (%)	*n* (%)	*n* (%)	*n* (%)	
Age (Mean, SD)	69.1 (7.0)	69.4 (7.0)	68.3 (7.2)	70.3 (6.9)	0.51
Years since initial diagnosis (Mean, SD)	5.9 (5.2)	5.9 (5.9)	6.1 (4.5)	5.7 (5.1)	0.94
Body Mass Index (Mean, SD)	30.3 (6.5)	31.2 (8.1)	29.6 (5.4)	29.7 (4.8)	0.47
Hispanic Ethnicity					0.16
No	100 (88.5)	37 (82.2)	41 (95.3)	22 (88.0)	
Yes	13 (11.5)	8 (17.8)	2 (4.7)	3 (12.0)	
Race					0.86
White	87 (77.0)	33 (73.3)	33 (76.7)	21 (84.0)	
Black and African American	14 (12.4)	6 (13.3)	6 (14.0)	2 (8.0)	
Other^b^	12 (10.6)	6 (13.3)	4 (9.3)	2 (8.0)	
Marital status					0.78
Single/Divorced/Separated/Widowed	26 (23.2)	9 (20.5)	10 (23.3)	7 (28.0)	
Married/Domestic Partnership	86 (76.8)	35 (79.5)	33 (76.7)	18 (72.0)	
Missing	1	1	.	.	
Education level					0.12
Less than high school/High school grad/GED	16 (14.3)	11 (24.4)	4 (9.5)	1 (4.0)	
Some college/Assoc. Degree/Vocational School	32 (28.6)	13 (28.9)	11 (26.2)	8 (32.0)	
Bachelor’s degree or higher	64 (57.1)	21 (46.7)	27 (64.3)	16 (64.0)	
Missing	1	.	1	.	
Income					0.69
<$30,000	11 (12.1)	5 (14.3)	5 (13.5)	1 (5.3)	
$30,000-$49,999	8 (8.8)	2 (5.7)	5 (13.5)	1 (5.3)	
$50,000-$69,999	14 (15.4)	7 (20.0)	4 (10.8)	3 (15.8)	
$70,000 or more	58 (63.7)	21 (60.0)	23 (62.2)	14 (73.7)	
Missing	22	10	6	6	
Health insurance					0.54
No	3 (2.7)	2 (4.4)	1 (2.3)	24 (100.0)	
Yes	109 (97.3)	43 (95.6)	42 (97.7)	1	
Missing	1	.	2		
Cancer diagnosis					0.12
Prostate only	87 (77.0)	36 (80.0)	30 (69.8)	21 (84.0)	
Prostate and other types of invasive cancer(s)	11 (9.7)	2 (4.4)	5 (11.6)	4 (16.0)	
Other cancer type(s)	15 (13.3)	7 (15.6)	8 (18.6)	.	
Stage of Disease					0.30
Local	73 (64.6)	37 (60.0)	38 (65.1)	8(72.0)	
Regional	10 (8.8)	2 (4.4)	6 (13.9)	2 (8.0)	
Distant/Biochemical Disease	30 (26.6)	16 (35.6)	9 (21.0)	5 (20.0)	
Currently taking hormone therapy					0.15
No	84 (74.3)	29 (64.4)	35 (81.4)	20 (80.0)	
Yes	29 (25.7)	16 (35.6)	8 (18.6)	5 (20.0)	

*TCG*, Tai Chi Qigong; *EIM*, Exercise Intensity Matched; *UC*, Usual Care; *SD*, Standard Deviation

^a^ Demographic and clinical characteristics of participants were compared among three arms using ANOVA and chi-square analysis

^b^ “Other” races include: 2 American Native or Alaska Native, 1 Chinese, 1 Indian, 1 Filipino, 1 multiracial, and 6 Hispanic participants did not specify their race

Participants in the TCQ and EIM arms showed no statistically significant differences in class attendance across 24 classes, with high average attendance rates of 78.4% and 76.8%, respectively. No significant differences were found in treatment expectancy and credibility between the two arms from baseline to 6-week mid-intervention and 1-week post intervention (*P* > 0.05). Average perceived exertion was in the light exertion range and there was not a clinically meaningful difference in perceived exertion between the TCQ (9.8 ± 2.1) and EIM (10.9 ± 2.2) arms [39].

**Fig. 2:**
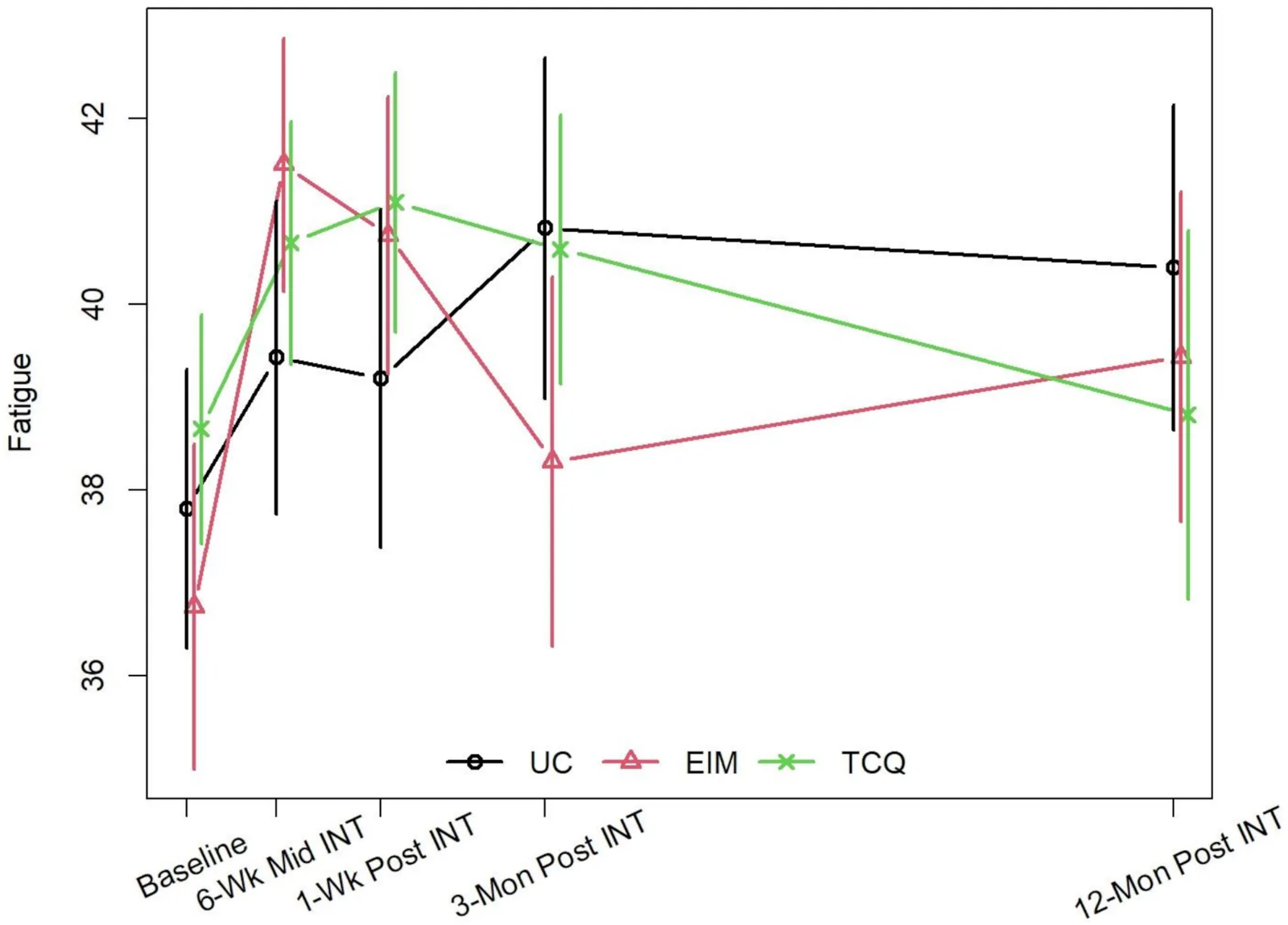
Changes in Fatigue from Baseline to 12-Month Follow-Up in the Tai Chi Qigong, Exercise Intensity Matched, and Usual Care Arms *TCG*, Tai Chi Qigong; *EIM*, Exercise Intensity Matched; *UC*, Usual Care; 6-Wk Mid INT, 6-Week Mid Intervention; 1-Wk post INT, 1-Week Post Intervention; 3-Mon Post INT, 3-Months Post Intervention; 12-Mon Post INT, 12-Months Post Intervention.

At the 3-month assessment, no clinically or statistically significant within-arm or between-arm differences in fatigue levels (i.e., FACIT scores) were observed (Table 2; Fig. 2, Supplemental Table 2). However, the TCQ and EIM arms showed some short-term within-arm improvement in fatigue at 1-week post-intervention, with the EIM arm showing greater improvement that appeared to be both clinically and statistically significant (mean change = 3.21; 95% CI [1.49, 4.92]) (Table 2). These improvements were consistently observed in two sensitivity analyses: one based on multiple imputation (Supplemental Table 3) and the other conducted only in participants with prostate cancer (*n* = 98, Supplemental Tables 4 and 5). At all timepoints, in the primary analysis (Table 2) and sensitivity analyses (Supplemental Tables 3, 4, and 5), no between-group differences in the change from baseline were observed in any of the pairwise comparisons (i.e., TCQ vs. EIM, TCQ vs. UC, EIM vs. UC). Participants were requested to complete daily physical activity logs; however, due to non-compliance we were not able to calculate the duration and frequency of home practice and other types of physical activity and determine if there were group differences in the frequency and duration of home practice. Finally, in an exploratory subgroup analysis comparing the effects of the interventions between patients receiving hormone therapy and those not undergoing this treatment (Supplemental Table 6, Supplemental Fig. 1), both the TCQ and EIM arms demonstrated appreciably greater improvements compared to the UC arm at the 6-week mid intervention and 1-week post-intervention assessments among patients receiving hormone therapy, although the improvements were generally not clinically or statistically significant.

**Table 2:** Changes in fatigue from baseline: linear mixed model analysis for within and Between-Arm comparison

	Baseline	6-week mid-intervention		1-week post-intervention		3-month post-intervention		12-month post-intervention	
	Mean (SE)	Mean (SE)	Change from baseline MD (95% CI)	Mean (SE)	Change from baseline MD (95% CI)	Mean (SE)	Change from baseline MD (95% CI)	Mean (SE)	Change from baseline MD (95% CI)
TCQ	38.7 (1.4)	39.9 (1.4)	1.24 (-0.36, 2.84)	40.3 (1.4)	1.69 (0.12, 3.26)^*^	39.8 (1.5)	1.16 (-0.55, 2.87)	38.0 (1.6)	-0.63 (-2.79, 1.54)
EIM	36.7 (1.4)	39.5 (1.5)	2.72 (0.95, 4.48)^*^	40.0 (1.5)	3.21 (1.49, 4.92)^*^	37.3 (1.5)	0.60 (-1.24, 2.44)	37.7 (1.7)	0.96 (-1.29, 3.21)
UC	37.8 (1.9)	39.1 (1.9)	1.33 (-0.84, 3.50)	39.4 (1.9)	1.62 (-0.55, 3.80)	39.1 (2.0)	1.34 (-1.15, 3.82)	39.1 (2.3)	1.28 (-1.85, 4.41)
TCQ vs. EIM			-1.48 (-3.86, 0.91)		-1.52 (-3.84, 0.80)		0.56 (-1.96, 3.07)		-1.59 (-4.71, 1.53)
TCQ vs. UC			-0.09 (-2.79, 2.61)		0.07 (-2.61, 2.75)		-0.18 (-3.19, 2.84)		-1.91 (-5.72, 1.90)
EIM vs. UC			1.38 (-1.41, 4.18)		1.59 (-1.18, 4.36)		-0.73 (-3.82, 2.36)		-0.32 (-4.18, 3.54)

*TCG*, Tai Chi Qigong; *EIM*, Exercise Intensity Matched Intervention; *UC*, Usual Care; *SD*, Standard Deviation.; *MD*, Mean Difference

*P < 0.05

## Discussion

In this trial involving men aged 55 and older with cancer, we did not find significant differences in improvement in fatigue between TCQ, EIM, or UC at the three-month follow-up in the overall sample and in the subgroup of men with prostate cancer. In secondary analysis, both the TCQ and EIM arms reported significant short-term, within-arm improvements in fatigue 1-week after the intervention was completed, whereas the improvements in the UC arm were not statistically significant. While the EIM showed clinically meaningful improvements in fatigue immediately post intervention, the improvement in the TCQ arm was not considered to be a clinically meaningful difference (> 3 points is considered clinically meaningful) [43–45]. No significant improvement in fatigue at 12 months was observed for any of the three study arms. However, in the exploratory subgroup analysis for hormone therapy status, TCQ and EIM appeared to have stronger but not clinically significant effects, especially at the 6-week mid-intervention and 1-week post-intervention assessments in men receiving hormone therapy during the intervention. However, in the exploratory subgroup analysis for androgen deprivation therapy status, TCQ and EIM appeared to have stronger but not clinically significant effects, especially at the 6-week mid-intervention and 1-week post-intervention assessments in men receiving hormone deprivation therapy during the intervention. This is potentially due to less variation in fatigue levels in this subgroup compared to men not on hormonal therapy [51].

Although the study’s findings suggest that TCQ and low intensity exercise result in short-term improvements in fatigue, these improvements were not sustained after cessation of program participation. These findings do not support the hypothesis that TCQ is superior to a low-intensity exercise program or usual care in improving levels of fatigue in older men with cancer. Several reasons might explain these findings. First, interruptions during the COVID-19 pandemic affected enrollment and retention of the trial, preventing us from reaching accrual goals and limiting the statistical power to detect significant reductions in fatigue. Second, COVID-19 pandemic-related stress, distress, sleep disturbance and reduced physical activity might also have caused fatigue [52], thereby attenuating the potential intervention effects that might otherwise have been observed. Third, based on the positive trends regarding short-term improvements in fatigue in the TCQ and EIM arms, a 12-week dose of two supervised sessions per week of TCQ or light exercise may have been insufficient to achieve durable effects after completing the group-based interventions. A higher intensity and/or ongoing engagement in a group-based program and higher dose or frequency of supervised TCQ may be necessary to achieve clinically meaningful improvements in fatigue in older male cancer survivors. In addition, men may benefit from ongoing home practice after the group-based intervention period and the combination of other intervention such as resistance training, which has also been shown to effectively alleviate or mitigate cancer-related fatigue [53, 54]. Most studies show that exercise interventions lose efficacy or have diminished effectiveness after the intervention is completed because sustained adherence to supervised and unsupervised exercise becomes more challenging even among motivated patients [55]. This suggests the need for effective behavioral support interventions to optimize long-term maintenance. Maintenance of TCQ and exercise following completion of the program may be especially challenging for older patients whose fitness levels may be poor, have transportation challenges to get to classes or are not able to afford classes. The unique needs of older patients should be considered when developing survivorship care plans.

Our null findings regarding the primary outcome are consistent with previous studies on the efficacy of TCQ in prostate cancer [33, 56] and breast cancer survivors [57, 58]. However, our findings differ from those of other previous studies in prostate and survivors with other types of cancers, where TCQ significantly reduced fatigue [57–62]. Substantial heterogeneity exists across previous studies [60]. Despite a shared emphasis on integrating traditional TCQ principles (e.g., gentle movements, synchronized breathing and meditation), the interventions varied in TCQ forms, duration, and frequency as well as length of follow-up. Commonly employed TCQ forms included Yang Style Tai Chi (10 to 24 forms) [33, 57–60], Chen Style Tai Chi [63], and Eight Pieces of Brocade (Ba Duan Jin) [58]. Intervention duration ranged from 5 weeks to 6 months, with frequencies from once to 7 times per week. Session lengths varied from 20 to 30 min to 2 h, with the majority lasting 1 h. Studies that utilized non-active control groups demonstrated greater effect sizes compared to those using active controls, such as light exercise or cognitive behavioral therapy [15, 59, 61]. This heterogeneity likely contributed to conflicting results. Nonetheless, our findings suggest that both TCQ and light intensity group-based exercise led to short-term improvements in fatigue in older male cancer survivors.

Fatigue in older adults can result from a combination of factors, including cancer and its treatments (e.g., androgen deprivation therapy), as well as chronic diseases such as cardiovascular disease, diabetes, and neurological disorders. Additionally, other common factors that contribute to fatigue in older adults are pain, anxiety and depression, stress, sleep disturbance, medications (e.g., antidepressants, cardiovascular drugs) and cognitive decline. While the distribution of common comorbidities was likely balanced across study arms due to randomization, we were unable to optimally measure all potential causes of fatigue. Potential mechanisms underlying CRF are disruptions in the central nervous system (hypothalamic-pituitary-adrenal access, inflammation) and/or the peripheral nervous system (e.g., disruptions in energy metabolism) system [64]. Although we were unable to disentangle the specific mechanisms underlying fatigue in this analysis, we plan to investigate whether TCQ, compared to EIM or UC, regulates pro- and anti-inflammatory cytokines and influences the expression of two major fatigue-associated functional gene clusters: (a) inflammation, vasodilation, and metabolite sensing, and (b) energy and adrenergic activation. These analyses will help clarify the biological mechanisms through which both TCQ and a low intensity exercise program may impact fatigue.

Our study has some limitations as well as strengths that should be acknowledged. First, given that we did not reach our accrual goal; thus, the analysis was underpowered to detect differences in fatigue between the study arms. Second, the participants were primarily White and non-Hispanic, and the majority were college-educated, thereby limiting the generalizability of findings. Third, male cancer survivors with cancer-related fatigue are a heterogenous group that includes individuals experiencing the influence of frailty, medications, and chronic health conditions. We did not examine these and other potentially treatable mechanisms but acknowledge that they should be considered in future research. Fourth, because of the relatively small sample size we were unable to perform meaningful subgroup analyses to determine if the intervention was effective for some subgroups and not others. Another limitation is that there were few participants who were very old (age 80 and older), a common limitation in studies of exercise and TCQ. Fifth, there were the previously mentioned effects of the global COVID-19 pandemic that overlapped with the study duration. Data on COVID-19 positive tests or the presence of long-COVID were not consistently available or reported, therefore we were not able to optimally evaluate the impact of COVID on fatigue in our study. Finally, some of the exclusion criteria were necessary because certain medications and health conditions interfere with the inflammation and gene expression biomarkers that will be analyzed. Our study’s results may not be generalizable to a wider population because of the extensive exclusion criteria.

Despite these limitations, our study had several noteworthy strengths. The study design was a three-armed trial that included both non-active and active control groups that focused on male cancer survivors, an understudied population in mind-body exercise research. Class attendance was high, and the exercise intensity and perceptions of treatment credibility and effectiveness of the two active study arms were similar.

In summary, despite high adherence and evidence for fidelity, we did not find that TCQ, compared with a usual care or EIM, significantly reduced fatigue in older male cancer survivors at 3 months. However, there were promising data suggesting short-term effectiveness of TCQ and light intensity exercise in improving fatigue. Larger trials of TCQ in male cancer survivors with CRF are needed before TCQ can be recommended for reducing fatigue in this population.

## Electronic supplementary material

Below is the link to the electronic supplementary material.

Supplementary Material 1

## Data Availability

The datasets used and/or analyzed during the current study are de-identified and available from the corresponding author on reasonable request.

## Abbreviations

ADT: Androgen-deprivation therapy
ASCO: American Society of Clinical Oncology
BMT: Body-mind training
BT: Body training
CRF: Cancer-related fatigue
EIM: Exercise intensity-matched
FACIT-F: Functional Assessment of Chronic Illness-Fatigue
FCS: Fully conditional specification multiple imputation
HERO: Health Recovery and Empowerment Outcomes
KPS: Karnofsky performance status score
NCCN: The National Comprehensive Cancer Network
PHQ-9: Patient Health Questionnaire-9
PROMIS Fatigue scale: Patient-Reported Outcomes Measurement Information System Fatigue scale
PSA: Prostate-specific antigen
SF-36 Vitality scale: Medical Health Outcome Study Short Form Vitality/Fatigue subscale
TCG: Tai Chi Qigong
UC: Usual care
