# Supplementary material for: Project HERO: a randomized trial of Tai Chi qigong versus intensity-matched exercise and usual care for fatigue in older male cancer survivors
Source: BMC Complement Med Ther. 2025 Jul 4;25:239. doi: 10.1186/s12906-025-04988-7 (PMC12228344; doi:10.1186/s12906-025-04988-7)
Supplement: Supplementary file 1 — Supplementary Material 1 [file 12906_2025_4988_MOESM1_ESM.docx]

**Supplementary Materials**

**S Table 1. Sociodemographic and Clinical Characteristics of Participants with Prostate Cancer Overall and by Study Arm (N = 98)**

| Study Arm | All  (N=98)  n (%) | TCQ (N=38)  n (%) | EIM (N=35)  n (%) | UC (N=25)  n (%) | P value^a^ |
| --- | --- | --- | --- | --- | --- |
| Age (Mean, SD) | 69.4 (7.0) | 69.5 (6.7) | 68.6 (7.0) | 70.3 (6.9) | 0.63 |
| Years since initial diagnosis (Mean, SD) | 6.1 (5.3) | 6.4 (6.2) | 6.0 (4.4) | 5.7 (5.1) | 0.87 |
| Body Mass Index (Mean, SD) | 30.2 (6.8) | 31.3 (8.6) | 29.4 (5.5) | 29.7 (4.8) | 0.44 |
| Hispanic Ethnicity  No  Yes | 86 (87.8)  12 (12.2) | 31 (81.6)  7 (18.4) | 33 (94.3)  2 (5.7) | 22 (88.0)  3 (12.0) | 0.25 |
|  |  |  |  |  |  |
| Race  White  Black  Other | 75 (76.5)  12 (12.2)  11 (11.2) | 28 (73.7)  5 (13.2)  5 (13.2) | 26 (74.3)  5 (14.3)  4 (11.4) | 21 (84.0)  2 (8.0)  2 (8.0) | 0.89 |
|  |  |  |  |  |  |
| Marital status  Single/Divorced/Separated/Widowed  Married/Domestic Partnership  Missing | 23 (23.7)  74 (76.3)  1 | 8 (21.6)  29 (78.4)  1 | 8 (22.9)  27 (77.1)  . | 7 (28.0)  18 (72.0)  . | 0.84 |
|  |  |  |  |  |  |
| Education level  Less than high school/High school grad/GED  Some college/Assoc. Degree/Vocational School  Bachelor’s degree or higher  Missing | 12 (12.4)  28 (28.9)  57 (58.8)  1 | 9 (23.7)  11 (28.9)  18 (47.4)  . | 2 (5.9)  9 (26.5)  23 (67.6)  1 | 1 (4.0)  8 (32.0)  16 (64.0)  . | 0.09 |
|  |  |  |  |  |  |
| Income  <$30,000  $30,000-$49,999  $50,000-$69,999  $70,000 or more  Missing | 9 (9.2)  7 (7.1)  13 (13.3)  51 (52.0)  28 | 4 (10.5)  2 (5.3)  6 (15.8)  18 (47.4)  8 | 4 (11.4)  4 (11.4)  4 (11.4)  19 (54.3)  4 | 1 (4.0)  1 (4.0)  3 (12.0)  14 (56.0)  6 | 0.81 |
|  |  |  |  |  |  |
| Health insurance  No  Yes  Missing | 3 (3.1)  94 (96.9)  1 | 2 (5.3)  36 (94.7)  . | 1 (2.9)  34 (97.1)  2 | 24 (100.0)  1 | 0.50 |
|  |  |  |  |  |  |
| Cancer diagnosis  Prostate only  Prostate and other types of invasive cancer(s) | 87 (88.8)  11 (11.2) | 36 (94.7)  2 (5.3) | 30 (85.7)  5 (14.3) | 21 (84.0)  4 (16.0) | 0.32 |
|  |  |  |  |  |  |
|  |  |  |  |  |  |
| Cancer stage |  |  |  |  | 0.25 |
| Localized | 59 (60.2) | 20 (52.6) | 21 (60.0) | 18 (72.0) |  |
| Regional | 9 (9.2) | 2 (5.3) | 5 (14.3) | 2 (8.0) |  |
| Distant | 30 (30.6) | 16 (42.1) | 9 (25.7) | 5 (20.0) |  |
| Currently taking hormone therapy  No  Yes | 69 (70.4)  29 (29.6) | 22 (57.9)  16 (42.1) | 27 (77.1)  8 (22.9) | 20 (80.0)  5 (20.0) | 0.09 |
|  |  |  |  |  |  |

*TCG,* Tai Chi Qigong; *EIM,* Exercise Intensity Matched; *UC,* Usual Care; *SD,* Standard Deviation.

^a^ The demographic and clinical characteristics of participants were compared among three arms using ANOVA and chi-square analysis.

**S Table 2. Raw Mean and Standard Deviation for Fatigue at All Timepoints (N = 113)**

|  | Baseline  Mean (SD) | 6-week mid-intervention  Mean (SD) | 1-week post-intervention  Mean (SD) | 3-month post-intervention  Mean (SD) | 12-month post-intervention  Mean (SD) |
| --- | --- | --- | --- | --- | --- |
|  |  |  |  |  |  |
| TCQ | 38.7 (8.3) | 40.7 (8.1)  41.5 (7.6)  39.4 (7.7) | 41.1 (8.7)  40.7 (8.4)  39.2 (8.2) | 40.6 (8.8)  38.3 (11.3)  40.8 (7.6) | 38.8 (11.3)  39.4 (9.7)  40.4 (6.8) |
| EIM | 36.7 (11.5) |  |  |  |  |
| UC | 37.8 (7.5) |  |  |  |  |

*TCG,* Tai Chi Qigong; *EIM,* Exercise Intensity Matched; *UC,* Usual Care; *SD,* Standard Deviation.

**S Table 3. Changes in Fatigue from Baseline: Linear Mixed Model Analysis for Within and Between-Arm Comparison Based on Multiple Imputation (N = 113)**

|  | Baseline | 6-week mid-intervention | | 1-week post-intervention | | 3-month post-intervention | | 12- month post-intervention | |
| --- | --- | --- | --- | --- | --- | --- | --- | --- | --- |
|  | Mean (SE) | Mean (SE) | Change from baseline  MD (95% CI) | Mean (SE) | Change from baseline  MD (95% CI) | Mean (SE) | Change from baseline  MD (95% CI) | Mean (SE) | Change from baseline  MD (95% CI) |
| TCQ | 38.7 (1.4) | 39.9 (1.5) | 1.26 (-0.45, 2.97) | 40.2 (1.4) | 1.56 (-0.07, 3.12)* | 39.8 (1.5) | 1.17 (-0.63, 2.98) | 37.7 (1.7) | -0.99 (-3.31, 1.33) |
| EIM | 36.7 (1.4) | 39.4 (1.6) | 2.54 (0.61, 4.46)** | 39.4 (1.5) | 2.68 (0.95, 4.42)** | 37.5 (1.6) | 0.79 (-1.24, 2.83) | 37.0 (1.8) | 0.29 (-2.25, 2.83) |
| UC | 37.8 (1.9) | 39.2 (2.0) | 1.40 (-0.96, 3.770) | 39.5 (1.9) | 1.70 (-0.44, 3.85) | 39.2 (2.1) | 1.38 (-1.28, 4.04) | 38.3 (2.3) | 0.53 (-2.68, 3.74) |
| TCQ vs. EIM |  |  | -1.28 (-3.71, 1.15) |  | -1.12 (-3.46, 1.22) |  | 0.38 (-2.32, 3.08) |  | -1.28 (-4.46, 1.90) |
| TCQ vs. UC |  |  | -0.15 (-3.10, 2.810) |  | -0.14 (-2.86, 2.58) |  | -0.20 (-3.36, 2.95) |  | -1.52 (-5.42, 2.39) |
| EIM vs. UC |  |  | 1.13 (-1.92, 4.19) |  | 0.98 (-1.78, 3.74) |  | -0.58 (-3.76, 2.60) |  | -0.24 (-4.23, 3.75) |

*TCG,* Tai Chi Qigong; *EIM,* Exercise Intensity Matched; *UC,* Usual Care; *SE,* Standard Error.; *MD*, Mean Difference.

*P < 0.10; **P < 0.05

**S Table 4. Changes in Fatigue from Baseline in Participants with Prostate Cancer: Linear Mixed Model Analysis for Within and Between-Arm Comparison (N = 98)**

|  | Baseline | 6-week mid-intervention | | 1-week post-intervention | | 3-month post-intervention | | 12-month post-intervention | |
| --- | --- | --- | --- | --- | --- | --- | --- | --- | --- |
|  | Mean (SE) | Mean (SE) | Change from baseline  MD (95% CI) | Mean (SE) | Change from baseline  MD (95% CI) | Mean (SE) | Change from baseline  MD (95% CI) | Mean (SE) | Change from baseline  MD (95% CI) |
| TCQ | 38.7 (1.5) | 40.9 (1.5) | 2.11 (0.45, 3.77)^**^ | 40.8 (1.5) | 2.04 (0.37, 3.70)^**^ | 39.9 (1.6) | 1.18 (-0.61, 2.97) | 39.4 (1.7) | 0.63 (-1.55, 2.82) |
| EIM | 37.1 (1.5) | 39.3 (1.6) | 2.18 (0.37, 3.99)^**^ | 40.1 (1.6) | 3.00 (1.17, 4.82)^**^ | 37.3 (1.6) | 0.18 (-1.73, 2.10) | 37.6 (1.8) | 0.49 (-1.79, 2.77) |
| UC | 37.8 (1.8) | 39.1 (1.9) | 1.33 (-0.74, 3.40) | 39.4 (1.9) | 1.62 (-0.54, 3.77) | 39.1 (2.0) | 1.32 (-1.13, 3.77) | 39.1 (2.2) | 1.27 (-1.68, 4.22) |
| TCQ vs. EIM |  |  | -0.07 (-2.53, 2.39) |  | -0.96 (-3.43, 1.51) |  | 1.00 (-1.62, 3.62) |  | 0.14 (-3.01, 3.30) |
| TCQ vs. UC |  |  | 0.78 (-1.87, 3.44) |  | 0.42 (-2.30, 3.14) |  | -0.14 (-3.17, 2.89) |  | -0.64 (-4.31, 3.03) |
| EIM vs. UC |  |  | 0.85 (-1.89, 3.60) |  | 1.38 (-1.44, 4.20) |  | -1.14 (-4.25, 1.97) |  | -0.78 (-4.51, 2.95) |

*TCG,* Tai Chi Qigong; *EIM,* Exercise Intensity Matched; *UC,* Usual Care; *SD,* Standard Deviation.; *MD*, Mean Difference.

*P < 0.10; **P < 0.05

**S Table 5. Changes in Fatigue from Baseline in Participants with Prostate Cancer: Linear Mixed Model Analysis for Within and Between-Arm Comparison Based on Multiple Imputation (N = 98)**

|  | Baseline | 6-week mid-intervention | | 1-week post-intervention | | 3-month post-intervention | | 12-month post-intervention | |
| --- | --- | --- | --- | --- | --- | --- | --- | --- | --- |
|  | Mean (SE) | Mean (SE) | Change from baseline  MD (95% CI) | Mean (SE) | Change from baseline  MD (95% CI) | Mean (SE) | Change from baseline  MD (95% CI) | Mean (SE) | Change from baseline  MD (95% CI) |
| TCQ | 38.7 (1.5) | 40.9 (1.6) | 2.16 (0.35, 3.98)^**^ | 40.6 (1.6) | 1.83 (0.04, 3.62)^**^ | 39.6 (1.6) | 0.87 (-1.01, 2.81) | 38.7 (1.8) | -0.08 (-2.68, 2.52) |
| EIM | 37.1 (1.5) | 39.5 (1.6) | 2.38 (0.61, 4.15)^**^ | 39.8 (1.6) | 2.66 (0.78, 4.54)^**^ | 37.5 (1.7) | 0.38 (-1.64, 2.39) | 37.5 (1.8) | 0.41 (-2.10, 2.92) |
| UC | 37.8 (1.8) | 39.3 (1.9) | 1.51 (-0.60, 3.61) | 39.7 (1.9) | 1.86 (-0.34, 4.06)^*^ | 38.9 (2.0) | 1.14 (-1.22, 3.49) | 38.5 (2.3) | 0.73 (-2.55, 4.01) |
| TCQ vs. EIM |  |  | -0.22 (-2.73, 2.30) |  | -0.83 (-3.45, 1.80) |  | 0.49 (-2.27, 3.25) |  | -0.49 (-4.12, 3.14) |
| TCQ vs. UC |  |  | 0.66 (-2.14, 3.46) |  | -0.03 (-2.79, 2.73) |  | -0.27 (-3.35, 2.81) |  | -0.81 (-4.86, 3.25) |
| EIM vs. UC |  |  | 0.87 (-1.82, 3.56) |  | 0.80 (-2.13, 3.72) |  | -0.76 (-3.85, 2.33) |  | -0.32 (-4.27, 3.64) |

*TCG,* Tai Chi Qigong; *EIM,* Exercise Intensity Matched; *UC,* Usual Care; *SD,* Standard Deviation.; *MD*, Mean Difference.

*P < 0.10; **P < 0.05

**S Table 6. Changes in Fatigue from Baseline: Linear Mixed Model Analysis for Within and Between-Arm Comparison by Hormone Therapy Status (N = 113)**

|  | Baseline | 6-week mid-intervention | | 1-week post-intervention | | 3-month post-intervention | | 12- month post-intervention | |
| --- | --- | --- | --- | --- | --- | --- | --- | --- | --- |
|  | Mean (SE) | Mean (SE) | Change from baseline  MD (95% CI) | Mean (SE) | Change from baseline  MD (95% CI) | Mean (SE) | Change from baseline  MD (95% CI) | Mean (SE) | Change from baseline  MD (95% CI) |
| *Receiving hormone therapy (N =29)* | | | | | | | | | |
| TCQ | 38.9 (2.2) | 41.8 (2.4) | 2.84 (-0.16, 5.84)^*^ | 41.8 (2.3) | 2.82 (0.13, 5.51)^**^ | 39.6 (2.3) | 0.68 (-1.93, 3.30) | 38.3 (2.7) | -0.63 (-4.56, 3.30) |
| EIM | 39.7 (2.0) | 42.4 (3.6) | 2.64 (-2.37, 7.65) | 42.0 (3.5) | 2.29 (-2.23, 6.91) | 40.6 (3.4) | 0.89 (-3.60, 5.39) | 42.0 (4.2) | 2.29 (-4.28, 8.87) |
| UC | 34.6 (3.9) | 34.0 (4.2) | -0.60 (-5.65, 4.47) | 32.4 (4.0) | -2.20 (-6.87, 2.47) | 33.4 (4.1) | -1.17 (-6.14, 3.80) | 32.3 (5.4) | -2.35(-10.68,5.99) |
| TCQ vs. EIM |  |  | 0.20 (-5.64, 6.03) |  | 0.52 (-4.82, 5.87) |  | -0.21 (-5.41, 4.99) |  | -2.92 (-10.58,4.74) |
| TCQ vs. UC |  |  | 3.44 (-2.44, 9.31) |  | 5.02 (-0.37, 10.40)^*^ |  | 1.85 (-3.76, 7.47) |  | 1.72 (-7.49,10.93) |
| EIM vs. UC |  |  | 3.24 (-3.87, 10.36) |  | 4.49 (-2.07, 11.06) |  | 2.06 (-4.64, 8.76) |  | 4.64 (-5.97,15.25) |
| *Not Receiving hormone therapy (N =84)* | | | | | | | | | |
| TCQ | 38.5 (1.8) | 38.8 (1.8) | 0.32 (-1.60, 2.97) | 39.4 (1.8) | 0.95 (-1.00, 2.89) | 40.0 (1.9) | 1.49 (-0.81, 3.80) | 38.0 (2.0) | -0.51 (-3.23, 2.21) |
| EIM | 36.1 (1.6) | 38.8 (1.6) | 2.77 (0.95, 4.58)^**^ | 39.5 (1.6) | 3.47 (1.65, 5.29)^**^ | 36.7 (1.7) | 0.64 (-1.45, 2.74) | 36.8 (1.8) | 0.74 (-1.61, 3.10) |
| UC | 38.6 (2.1) | 40.6 (2.2) | 1.97 (-0.39, 4.33) | 41.5 (2.2) | 2.91 (0.47, 5.35)^**^ | 40.8 (2.3) | 2.21 (-0.76, 5.17) | 41.0 (2.5) | 2.41 (-0.94, 5.75) |
| TCQ vs. EIM |  |  | -2.45 (-5.09, 0.19)^*^ |  | -2.52 (-5.19, 0.14)^*^ |  | 0.85 (-2.27, 3.97) |  | -1.26 (-4.86, 2.34) |
| TCQ vs. UC |  |  | -1.65 (-4.69, 1.39) |  | -1.97 (-5.09, 1.15) |  | -0.72 (-4.47, 3.04) |  | -2.92 (-7.23, 1.39) |
| EIM vs. UC |  |  | 0.80 (-2.18, 3.77) |  | 0.56 (-2.49, 3.60) |  | -1.56 (-5.20, 2.07) |  | -1.66 (-5.75, 2.43) |

*TCG,* Tai Chi Qigong; *EIM,* Exercise Intensity Matched; *UC,* Usual Care; *SE,* Standard Error.; *MD*, Mean Difference.

*P < 0.10; **P < 0.05

**S Figure 1.** **Change in Fatigue from Baseline to 12-Month Follow-Up in The Tai Chi Qigong, Exercise Intensity Matched, and Usual Care Arms by Hormone Therapy Status**


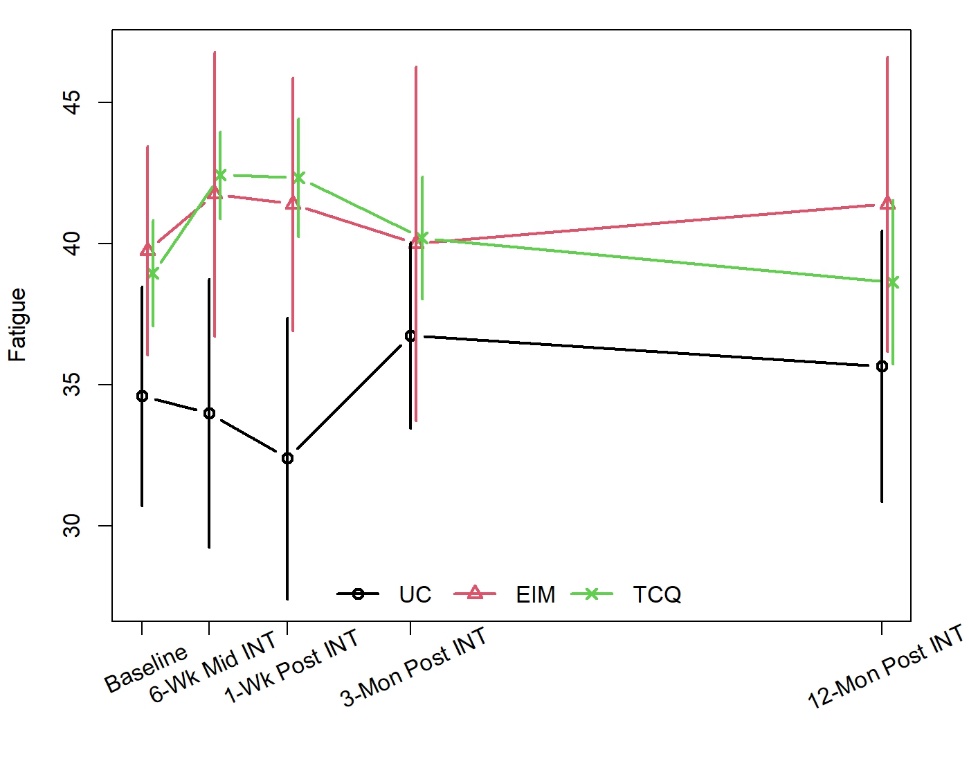

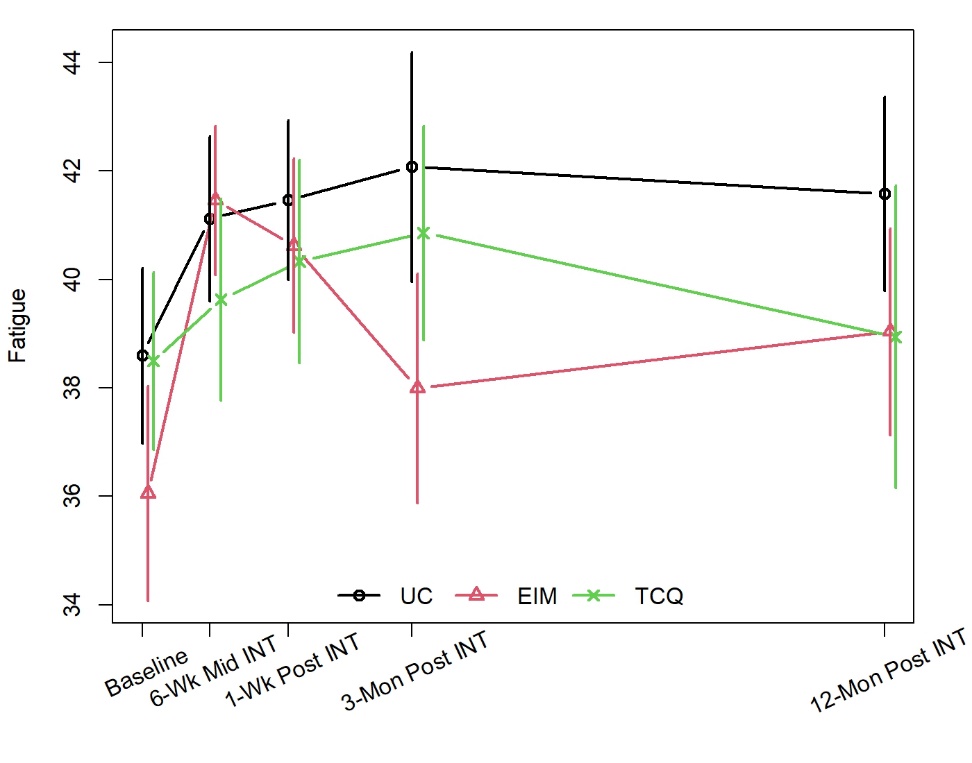


(a) (b)

(a) Changes in fatigue from baseline to 12-month follow-up among patient receiving hormone therapy

(b) Changes in fatigue from baseline to 12-month follow-up among patient not receiving hormone therapy

*TCG,* Tai Chi Qigong; *EIM,* Exercise Intensity Matched; *UC,* Usual Care; 6-Wk Mid INT, 6-Week Mid Intervention; 1-Wk post INT, 1-Week Post Intervention; 3-Mon Post INT, 3-Months Post Intervention; 12-Mon Post INT, 12-Months Post Intervention.
